# Update on Statin Treatment in Patients with Neuropsychiatric Disorders

**DOI:** 10.3390/life11121365

**Published:** 2021-12-08

**Authors:** Razieh Avan, Adeleh Sahebnasagh, Javad Hashemi, Mahila Monajati, Fatemeh Faramarzi, Neil C. Henney, Fabrizio Montecucco, Tannaz Jamialahmadi, Amirhossein Sahebkar

**Affiliations:** 1 Department of Clinical Pharmacy, Medical Toxicology and Drug Abuse Research Center (MTDRC), School of Pharmacy, Birjand University of Medical Sciences, Birjand 9717853577, Iran; avanr91@gmail.com; 2 Clinical Research Center, Department of Internal Medicine, North Khorasan University of Medical Sciences, Bojnurd 9453155166, Iran; masoomehsahebnasagh@gmail.com; 3 Department of Pathobiology and Laboratory Sciences, School of Medicine, North Khorasan University of Medical Sciences, Bojnurd 9453155166, Iran; j.hashemii@yahoo.com; 4 Department of Internal Medicine, Golestan University of Medical Sciences, Gorgan 4934174515, Iran; mahilamonajati@gmail.com; 5 Clinical Pharmacy Research Center, Iran University of Medical Sciences, Tehran 1445613131, Iran; faramarzif1984@gmail.com; 6 Pharmacy & Biomolecular Sciences, Liverpool John Moores University, Liverpool L3 5UX, UK; N.C.Henney@ljmu.ac.uk; 7 IRCCS Ospedale Policlinico San Martino Genoa-Italian Cardiovascular Network, 10 Largo Benzi, 16132 Genoa, Italy; fabrizio.montecucco@unige.it; 8 First Clinic of Internal Medicine, Department of Internal Medicine, University of Genoa, 6 Viale Benedetto XV, 16132 Genoa, Italy; 9 Department of Nutrition, Faculty of Medicine, Mashhad University of Medical Sciences, Mashhad 9177948564, Iran; jamiat931@gmail.com; 10 Applied Biomedical Research Center, Mashhad University of Medical Sciences, Mashhad 9177948564, Iran; 11 Biotechnology Research Center, Pharmaceutical Technology Institute, Mashhad University of Medical Sciences, Mashhad 9177948954, Iran; 12 Department of Biotechnology, School of Pharmacy, Mashhad University of Medical Sciences, Mashhad 9177948954, Iran

**Keywords:** statins, neuropsychiatric disorders, clinical trials, adverse effects, major depressive disorder, schizophrenia, anxiety, obsessive-compulsive disorder, bipolar disorder, delirium, autism spectrum disorders

## Abstract

Statins are widely accepted as first-choice agents for the prevention of lipid-related cardiovascular diseases. These drugs have both anti-inflammatory and anti-oxidant properties, which may also make them effective as potential treatment marked by perturbations in these pathways, such as some neuropsychiatric disorders. In this narrative review, we have investigated the effects of statin therapy in individuals suffering from major depressive disorder (MDD), schizophrenia, anxiety, obsessive-compulsive disorder (OCD), bipolar disorder (BD), delirium, and autism spectrum disorders using a broad online search of electronic databases. We also explored the adverse effects of these drugs to obtain insights into the benefits and risks associated with their use in the treatment of these disorders. Lipophilic statins (including simvastatin) because of better brain penetrance may have greater protective effects against MDD and schizophrenia. The significant positive effects of statins in the treatment of anxiety disorders without any serious adverse side effects were shown in numerous studies. In OCD, BD, and delirium, limitations, and contradictions in the available data make it difficult to draw conclusions on any positive effect of statins. The positive effects of simvastatin in autism disorders have been evaluated in only a small number of clinical trials. Although some studies showed positive effect of statins in some neuropsychiatric disorders, further prospective studies are needed to confirm this and define the most effective doses and treatment durations.

## 1. Introduction

Statins (3-hydroxy-3-methylglutaryl coenzyme A reductase inhibitors) are categorized as first-choice agents for primary and secondary prevention of cardiovascular diseases (CVDs). These drugs reduce the circulating plasma concentration of cholesterols via inhibition of the main enzyme involved in cholesterol synthesis in the liver. Statins also suppress the inflammatory response and decrease the expression of lipopolysaccharide (LPS)-induced monocyte tissue factor [1]. Since these agents inhibit the production of mevalonate (MVA), which is responsible for cholesterol synthesis and several non-steroidal isoprenoid compounds, they are described as having “pleiotropic effects” [2], which makes these drugs distinct from most of the other lipid-lowering therapies [3,4]. These effects include improvements in endothelial function, thrombosis, plaque-stabilizing effects, vascular inflammation, and oxidation [5,6,7,8,9,10].

Observational findings have identified a relationship between statin use and decreased risk of fracture in osteoporosis patients. Several studies have also hypothesized a delay in the pathogenesis of Alzheimer’s disease, progression of aortic stenosis, valvular calcification, and congestive heart failure [11]. The anti-inflammatory effects of statins can be seen via decreased levels of C-reactive protein (CRP) and low-density lipoprotein (LDL) cholesterol. Statins also decrease tumor necrosis factor alpha (TNF-α) and gamma interferon (IFNγ) production in stimulated T cells, and reduce immune activations of T-helper cells [12]. Because of these effects, statins may also have therapeutic benefits in the treatment of psychiatric diseases, including major depressive disorder (MDD), schizophrenia, and dementia, which may be marked by perturbations in inflammation and immune system pathways [13,14,15]. For example, one study found that long-term use of statins was associated with a reduced risk of MDD in patients with coronary artery disease [16]. In addition, the negative symptoms of schizophrenia may be partially alleviated with adjuvant therapy of statins [17]. Furthermore, statin treatment may offer a protective effect in patients at risk of dementia, although unfortunately, these findings are not consistent [18,19,20,21].

Statins are divided into lipophilic or hydrophilic types based on their permeability across the blood-brain barrier (BBB). The hydrophilic statins, including pravastatin, rosuvastatin, and fluvastatin, have low BBB permeability. In contrast, lipophilic statins, such as simvastatin, lovastatin, and atorvastatin, can traverse the BBB more readily. The lipophilic statins can enter cells and become extensively distributed in different tissues via passive diffusion, whereas the hydrophilic statins more specifically target the liver [12]. Despite their diversity in lipophilicity and pharmacokinetics, the two classes of statins have relatively similar biological activities [22]. It is unclear whether or not some of the effects of statins are related to their brain penetration or mediated by cytokine reduction in the periphery, as found in the case of rosuvastatin [23]. As stated above, perturbations in inflammatory pathways have been implicated in the pathogenesis of many psychiatric and neurodegenerative diseases. For example, an abnormal inflammatory response could result in resistance to treatment in schizophrenia [24]. Therefore, many investigators have evaluated statin treatment as a potential means of reducing symptoms in mental health disorders [12].

**Figure 1:**
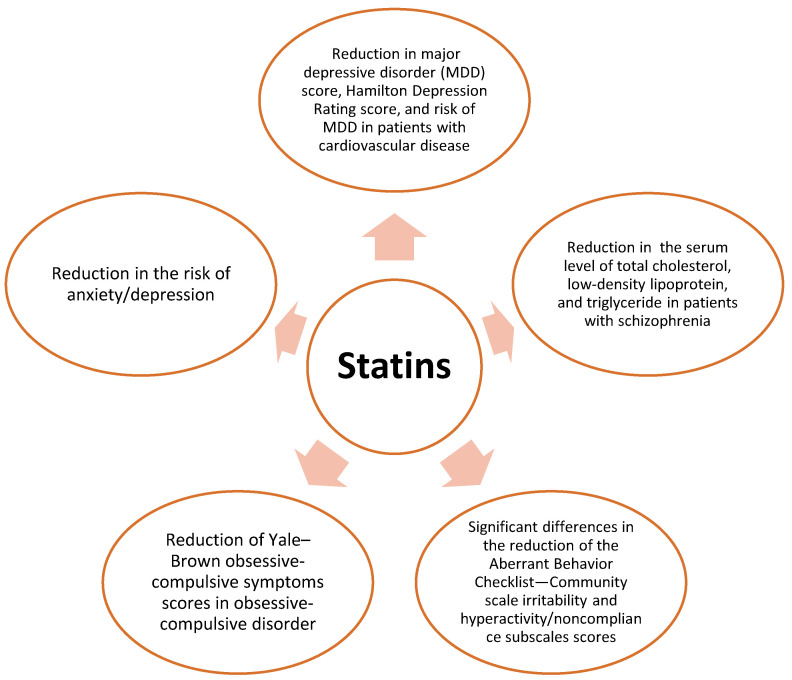
The effect of statins in neuropsychiatric disorders. Statins have been associated with the reductcion in major depressive disorders, improvement of lipid profile in schizophrenia, reduction of aberrant behavior, reduction of obsessive-compulsive symptoms and risk of anxiety/depression.

In this narrative review, we have explored clinical trials of statins in different neuropsychiatric diseases, including MDD, anxiety, bipolar disorder (BD), obsessive compulsive disorder (OCD), schizophrenia, panic disorder, autism, and delirium. The effect of statins in different neuropsychiatric disorders was illustrated in Figure 1.

## 2. Pharmacological Mechanism of Statins in Neuropsychiatric Disorders

There is a reasonable body of evidence that both central and peripheral inflammatory mechanisms may play a role in the etiology and/or the pathophysiology of many neuropsychiatric disorders, including MDD, BD, schizophrenia, and autism. Here, the inflammatory effects might be related to alterations in cytokines and acute-phase reactants. Inflammation in the nervous system is typically associated with microglial activation [25]. In the M1 type microglial activation, microglial cells may synthesize proinflammatory molecules, such as interleukin-1beta (IL-1β), TNF-α, and interleukin-6 (IL-6), superoxide radicals, glutamate, and nitric oxide (NO) [26,27]. Because of their role in neuronal signaling and signal transduction, a change in morphology of microglial cells, or in their number, is associated with the cognitive and behavioral symptoms reported in many psychiatric disorders [28]. The involvement of peripheral inflammation in psychiatric diseases was reviewed recently, and the tryptophan-kynurenine metabolic pathway is gaining growing attention. Pro-inflammatory molecules stimulated the indoleamine 2,3-dioxygenases in peripheral blood monocytes, which converts tryptophan to kynurenine. Suppression of this pathway may consequently be a significant factor in neuroprotection [29]. The most well-known mechanism of action of statins is through their effects on lowering endogenous cholesterol biosynthesis through inhibition of β-Hydroxy β-methylglutaryl-CoA (HMG-CoA) reductase, the rate-limiting enzyme in the pathway that produces cholesterol. However, considering the potential for treating psychiatric disorders the anti-inflammatory and anti-oxidant effects of statins are probably more important [12] although the action of statins in reducing LDL, which has pro-inflammatory activity, is certainly one means of reducing inflammation. In addition, statin treatment can directly reduce inflammation through inhibiting production of TNF-α and IFNγ, the T-helper cell immune response [30], and CRP release [31].

## 3. MDD and Statins

There is a growing body of evidence of a close relationship between inflammation and pathogenesis of MDD, and some anti-inflammatory medications are potential methods for alternative treatment of MDD. Therefore, the effects of anti-inflammatory agents on depressive disorders have been investigated in various studies including randomized controlled trials (RCTs) [12]. To date, several drugs with anti-inflammatory effects have shown some antidepressant effects, including non-steroidal anti-inflammatory drugs (NSAIDs), such as aspirin [32,33], celecoxib [34,35], monoclonal antibodies [36], minocycline [37], N-acetylcysteine [38], pioglitazone [39], and statins [12]. The direct anti-inflammatory effects of statins [40] have naturally led to studies exploring the potential impact of statins on MDD.

The standard first-line pharmacological treatment for MDD is long-term administration of a selective serotonin reuptake-inhibitor (SSRI) [41,42]. Due to the considerable role of inflammation in the pathophysiology of MDD [43,44,45], the concomitant treatment with statins and SSRI has been investigated compared to an SSRI alone [46]. In a nationwide cohort study on n = 872,216 Danish patients who were identified as SSRI users (1997–2012), it was shown that combinational treatment of a statin and an SSRI (n = 113,108) could reduce by 30% the risk of both psychiatric hospital contacts (for any reason) and psychiatric hospital contacts due to MDD compared to treatment with an SSRI alone. Importantly, there was no detrimental effect on all-cause mortality with concomitant use of SSRIs and statins compared with SSRI treatment alone. The estimation of treatment duration for both SSRIs and statins was performed on the basis of the prescribed number and dose of oral dosage forms and the Defined Daily Dose (DDD). The estimated treatment duration for each prescription was calculated as follows: treatment duration (days) = ((number of packages × number of tablets per package × dose of tablets)/DDD) × 1.15 + 7) to more closely align with actual drug consumption in clinical practice [46,47].

The potential effects of statins on mood and mood-related quality of life as a primary or secondary outcome have been investigated in numerous RCTs [48,49,50] and observational studies [16,51,52,53,54,55] with mixed outcomes. Taking a slightly different perspective, the effect of statin treatment on the risk of MDD remains unclear [56]. Two meta-analyses have shown that statins can be useful for reducing depressive symptoms in specific populations. In one meta-analysis of seven observational studies (four cohort, two nested case-control, and one cross-sectional study), it was found that depressive symptoms were less likely in statin users than non-users [53]. Another meta-analysis of three double-blind RCTs including 165 participants with moderate to severe MDD has shown that statins (lovastatin, atorvastatin, and simvastatin), as an add-on treatment, largely improved depressive symptoms although this outcome is limited by the small number of studies and short-term follow-up periods (6–12 weeks) [46].

The efficacy of statins in treating MDD has been studied in several RCTs [57,58,59]. For instance, a double-blind RCT conducted over six weeks in patients with untreated depressive symptoms after a coronary artery bypass graft (n = 46) concluded that simvastatin (20 mg/day) tended to improve depressive symptoms earlier and more effectively than did atorvastatin (20 mg/day) although the response rates were not significantly different [60]. If there is any positive effect from simvastatin, it may be linked to the ability of simvastatin to permeate the BBB. Another double-blinded RCT of Iranian patients (n = 60) who suffered from MDD included two intervention groups, one treated with citalopram 40 mg/day and atorvastatin 20 mg/day or the placebo group treated with citalopram 10 mg/day and placebo. This showed that atorvastatin had a greater effect on depressive symptoms than placebo. The results indicate that adding atorvastatin to a treatment regimen favorably influences depressive symptoms in patients with severe MDD [58]. In a randomized double-blind placebo controlled-clinical trial on 68 Iranian patients with MDD in the year 2013, the sample was randomly allocated into the treatment group (fluoxetine up to 40 mg/day plus lovastatin 30 mg/day) and the placebo group (in which patients took fluoxetine plus placebo). There was a significant decrease in MDD score in both groups based on the Hamilton Depression Rating (HDR) scale. However, there was a greater decrease in MDD score for the treatment group over that of the placebo group [57]. In a double-blind randomized placebo-controlled trial ran over six weeks, Gougol and coworkers compared one group taking fluoxetine (20 mg/day rising to 40 mg/day) plus simvastatin (20 mg/day) with individuals taking fluoxetine plus placebo. The results showed that the patients who received simvastatin experienced significantly early improvement and response rates with more reductions in HDR scores compared to the placebo group by the end of the trial [59]. The protocol of Youth Depression Alleviation: Augmentation with an anti-inflammatory agent (YoDA-A) was investigated by Quinn et al. in a 12-week triple-blinded placebo-controlled randomized trial on Australian 15–25-year-old participants with moderate to severe MDD in 2017. Participants received 10 mg/day rosuvastatin, or 100 mg/day aspirin, or placebo in addition to their usual other treatments. This study has identified the potential of new adjunctive treatment options included aspirin and rosuvastatin for youth MDD [61]. A summary of these trials is given in Table 1.

The association between statin use and reduced risk of MDD was reported in four prospective studies including large populations (n = 26,852–4,607,990) [47,62,63,64]. Statin use appears not to be associated with worsening of MDD in acute myocardial infarction (AMI) patients [65] or with any worsening of MDD risk in a community population (n = 1631) [66]. Moreover, the potential beneficial effects of statins on MDD have been reported in a number of community studies [47,62,63], which included larger numbers of participants and generally younger populations [66]. The beneficial influence of statins was observed in a non-randomized, one-year prospective study of patients experiencing symptoms of MDD following acute coronary syndrome (ACS), independent of medical status and the use of escitalopram. The combined treatment of statins and escitalopram was associated with greater effects than either drug alone. The study also demonstrated that lipophilic statins may have greater anti-depressive activity than hydrophilic statins [67]. The association between statin treatment and MDD was investigated in a large propensity score matched study. The results of this study did not find direct beneficial effects of statins on the risk of developing MDD. Instead, the findings indicated that statin users and non-users are probably equally likely to develop MDD but that MDD is more likely to be diagnosed and then treated among statin users [56]. A retrospective cohort study on U.S. adult patients who were initiated on lipophilic statin therapy (included atorvastatin, lovastatin and simvastatin) compared to hydrophilic statin therapy (included pravastatin and rosuvastatin) was performed to evaluate the risk of new-onset MDD. Of the participants who received lipophilic statins, only simvastatin was associated with a moderate increase in the risk of MDD. Altogether, lipophilic statin use, however, was not associated with a significant increase in the risk of incident MDD [68]. Since the beneficial anti-inflammatory effects of statins have not been seen in healthy populations without inflammatory loading, the direction of risks and beneficial associations of statins may depend on the characteristics of the participants. On the other hand, patients who experienced excess inflammation due to physical diseases, such as CVD, have shown reduced risk of MDD after using statins [12]. Further research is needed to determine whether lipophilic statins have any advantage over hydrophilic statins or vice versa in preventing or treating MDD. However, one might postulate that lipophilic statins (such as simvastatin) may have greater protective effects against MDD because of their ability to better penetrate the brain [62].

In summary, statins have been shown to reduce the risk of MDD in patients with an inflammatory condition, such as CVD, in both epidemiological and interventional studies. Statins show potential for improving depressive symptoms when used in combination with antidepressants, over and above either drug used alone.

## 4. Schizophrenia and Statins

Schizophrenia is a main cause of psychosis. Antipsychotics that antagonize monoamine receptors such as dopamine receptors are the common treatment options of the disease [69]. Some studies have shown the role of immune dysfunction and inflammation in the pathogenesis of schizophrenia [70,71]. The level of pro-inflammatory cytokines, such as IL-6, IL-1β, and TGF-β, is increased in patients [72,73]. A higher microglia activation is seen in the brain of patients with schizophrenia compared to the general population [74]. Therefore, the use of anti-inflammatory drugs as an adjuvant therapy may be useful in the treatment of schizophrenia. Metabolic syndrome and hyperlipidemia are typical disorders in patients with schizophrenia. These patients have a higher risk of developing CVD [75,76]. Hyperlipidemia is associated with the use of antipsychotic drugs, such as olanzapine and quetiapine. However, hyperlipidemia can, of course, be found in patients with no history of antipsychotic therapy. It is believed that hyperlipidemia develops because of the pathophysiology of schizophrenia [77,78]. Therefore, the use of anti-inflammatory cholesterol-lowering drugs, such as statins, could be effective in the treatment of schizophrenia symptoms. Hert et al. in a clinical trial showed that the use of daily rosuvastatin for three months decreased total cholesterol (TC), LDL, and triglyceride (TG) concentrations in patients with schizophrenia and schizoaffective disorder [79]. Ojala and coworkers showed that the use of statins as an add-on therapy could also reduce hyperlipidemia in psychiatric patients. This study evaluated the effects of several statins, including atorvastatin (19 ± 8.5 mg/day), fluvastatin (80 mg/day), rosuvastatin (10 mg/day), and simvastatin (10–20 mg/day), for up to two months. The authors showed that the use of statins along with second-generation antipsychotic agents could reduce TC, LDL, and TG [80]. Chaudhry et al. showed that the use of simvastatin (20 mg once daily rising to 40 mg once daily after four weeks) decreased the negative symptoms of schizophrenia after 26 weeks. No adverse effects were reported [81]. Mansi and colleagues in a retrospective cohort study showed that the use of statins did not decrease the risk of developing psychologic disorders including schizophrenia, psychosis, MDD, and BD in a military population [82]. In a study by Ghanizadeh and colleagues, 20 mg/day lovastatin was used for eight weeks as adjunctive therapy in the treatment of schizophrenia. However, no changes were observed in the Positive and Negative Syndrome Scale (PANSS) score between the intervention and placebo groups. In this study, no serious adverse events were observed in either group [83]. Similar outcomes were observed in the study of Vincenzi and coworkers in which they showed that pravastatin, as adjunctive therapy, lowers TC and LDL levels but did not change cognition or psychopathology of the disease in patients. Muscle soreness was the only notable side effect in the patients receiving pravastatin in comparison to the placebo group [84]. Tajik-Esmaeeli et al. in a double-blind RCT showed that the use of 40 mg daily simvastatin for eight weeks could reduce the negative but not positive symptoms of schizophrenia [17]. Osborn et al. showed that the use of statins did not reduce TC or decrease the risk of CVD after 12 months. However, this cluster RCT showed that the use of statins is associated with fewer psychiatric admissions and potential cost-effectiveness [85]. Overall, the balance of these studies shows that the use of statins could decrease the serum level of TC, LDL, and TG in patients with schizophrenia. A summary of these trials is given in Table 2. A meta-analysis by Nomura et al. also showed that statins have considerable potential as adjuvant therapy in reducing some of the symptoms of schizophrenia [86]. However, there is doubt in the beneficial effects of statins in reducing the psychiatric symptoms of the patients, which was also shown in a meta-analysis by Çakici et al. [87].

Some antipsychotic drugs may have an interaction with statins, as they have anti-inflammatory actions [88]. In addition, the lipophilic statins may be more effective due to their ability to pass through BBB. Simvastatin has been most prominently used in RCTs, and this is a lipophilic statin [68]. It seems that statin therapy is more effective in stabilized patients with schizophrenia. As animal studies have showed that a high dose of simvastatin is effective in the reduction of anxiety-like behavior by upregulating the N-methyl-D-aspartate (NMDA) receptor [89,90], it is suggested that a higher dose of statins may be more effective in human clinical trials. A review by Kim et al. recommended the use of statins at a sufficient dose for at least 12 weeks for a fairer test in these patients [12].

## 5. Anxiety Disorders and Statins

Statins are the most effective drugs for reducing plasma cholesterol and preventing cardiovascular events. Despite the effects on LDL, statins reduce CRP and subsequently lead to a reduction in pro-inflammatory cytokines interleukin-1 (IL-1), IL-6, TNF-α, cyclooxygenase (COX)-2, prostaglandin E2 (PGE2), reactive oxygen species (ROS), and NO. All statins have been shown to reduce the levels of inflammatory mediators, especially CRP levels [91,92,93,94]. The anti-inflammatory and anti-oxidant effects of statins may predict beneficial mechanisms in the treatment of various psychiatric diseases [95]. For example, long-term use of statins in coronary artery disease (CAD) patients reduces the risk of MDD, anxiety, and hostility [16]. Although beneficial effects of statins on anxiety disorders have been reported, the exact mechanism has not yet been determined. The results of several previous studies show that inflammation has an important effect on the pathogenesis of psychiatric diseases, such as anxiety-related disorders [96,97,98,99]. It has been suggested that statins in combination with conventional psychiatric medications may be useful in treating these disorders [12]. Chronic use of statins has also been reported to impair the function of neurotransmitter receptors, such as the 5-HT1A receptor. This suggests that statins can alter synaptic transmission by changing the function of neurotransmitter receptors in the brain [100]. The association between anxiety and non-adherence to statins was investigated in a prospective cohort study of n = 1924 individuals in 2016. Based on the results of the study, somatic anxiety-related symptoms but not psychological symptoms were found to be associated with non-adherence to statin therapy [101]. In another cohort study of n = 1632 subjects from January 1996 to December 2012, the incidence rate of anxiety/MDD in the head and neck cancer patients with a history of hyperlipidemia was markedly higher than in patients without hyperlipidemia. Statins had a protective effect for new-onset anxiety/MDD and decreased the risk of these disorders in patients with hyperlipidemia [102]. A retrospective cohort study in 2019 investigated the effects of statins on anxiety and MDD in patients with asthma–chronic obstructive pulmonary disease overlap syndrome (ACOS). The results showed that statin use significantly reduced the risks of anxiety and MDD [103]. A summary of these trials is given in Table 3. Alprazolam-like anxiolytic effects have also been reported for atorvastatin and simvastatin in animal models [104]. On the other hand, it has also been shown that chronic use of simvastatin, lowering cholesterol levels and decreasing the serotonin neurotransmission in the brain, leads to increased levels of anxiety in experimental animals [105].

Based on the results of the studies mentioned above, we conclude that statins have significant positive effects in the treatment of anxiety disorders, while no major adverse effects were observed. However, more large-scale, longer-term studies are needed to conclude whether the effects of statins on anxiety disorders provide sufficient evidence for active interventions.

## 6. OCD and Statins

OCD, which has a prevalence of 2–3% in the general population and 10% in psychiatric patients, is known for the uncontrollable repeating thoughts (obsessions) and behaviors (compulsions) in affected patients [106]. The first-line treatment of OCD is the use of SSRIs and sometimes clomipramine [107,108]. The etiology of the disease is unknown; however, studies have shown the connection between OCD and the increase in the dopamine neurotransmitter in basal ganglia [109]. An increase in glutamate may also be linked to OCD [110]. It has been shown that 40% of patients with OCD do not respond to first-line treatment options [111], and therefore, there is a need for a new approach to reduce the symptoms of the disease. As described above, statins have anti-inflammatory and neuroprotective properties [112,113]. Studies have shown that statins have an influence on the dopamine neurotransmitter and decreases dopamine concentrations in the prefrontal cortex [114,115]. Statins also affect the neurotransmitter glutamate. It has been shown that high doses of simvastatin maintain NMDA and inhibits neuronal degeneration in a Parkinson’s model [89]. Furthermore, statins have an influence on NO, an important neurotransmitter involved in defensive reactions, anxiety, and MDD [116,117,118]. It has been demonstrated that statins alter NO synthesis from endothelial cells [119], and this is important because NO also regulates dopamine and glutamate activity [119,120]. Therefore, the use of statins may decrease the pathogenic role of NO and other neurotransmitters in OCD patients.

Only a small number of studies have evaluated the use of statins in OCD patients in clinical trials. In a double-blind trial, Rahim and Sayyah showed that the use of 10 mg/day atorvastatin in OCD patients for 12 weeks significantly reduced the Yale–Brown obsessive-compulsive symptoms (Y-BOCS) obsession score. However, no significant difference in the Y-BOCS compulsive score was shown between the intervention and control groups. A significant decrease in libido was noted in the intervention group [121]. In another study, Akouchekian and colleagues showed that daily 40 mg atorvastatin reduced Y-BOCS scores after the fourth and eighth week of treatment of OCD patients. In this study, three patients were excluded due to severe drug reactions (such as headache, abdominal pain, and constipation) and therefore refused to continue [122]. A summary of these trials is given in Table 4. Although these studies demonstrated the effectiveness of atorvastatin in reducing Y-BOCS scores, due to the limitations of these studies, it is difficult to confirm the efficacy of atorvastatin in the treatment of patients with OCD.

## 7. BD and Statins

### 7.1. BD Definition

BD is a chronic progressive illness characterized by recurrent cycling episodes of mania and MDD. Symptoms of manic episodes include hyper-elevated mood, irritability, increased goal-directed activity, inflated self-esteem, poor judgment, and excessive motor activity. Depressive episodes of BD share the same characteristics as for major depressive disorder, including depressed mood, loss of interest in normally enjoyable activities, loss of self-worth, diminished ability to concentrate, and suicidal thoughts. Although the diagnostic criteria require there to be cycles mania/hypomania and MDD, the depressive phase represents the predominant mood state for both BD type I and BD type II. The pathophysiology of BD is poorly understood although treatment with drugs that target monoamine neurotransmitters sometimes provide relief. Mood stabilizers, including valproate, lithium, or atypical antipsychotics, are appropriate first-line treatments for acute mania. However, the neurobiology of BD remains unclear. Reviews report conflicting evidence for the effect of statins on mood [123]. Some studies support the association between statin use and adverse effects on mood, and some find that statin use reduces the risk of MDD [124].

### 7.2. Evidence from Clinical Trials

Lipids play an important role in brain functioning and are critical in the formation of neuronal cell membranes, the myelin sheath, and neuronal synapses. It has been hypothesized that decreased serum lipid levels in the brain can reduce available cholesterol, which can in turn decrease serotonergic activity through a reduction in 5-HT receptor expression. This could result in the alteration of behavior and psychological effects including irritability/aggression, anxiety or depressed mood, violent ideation or behavior, sleep disturbance, and suicide [125]. Simvastatin and lovastatin have a lipophilic structure that, compared to other statins, enables them to better cross the blood-brain barrier [126]. The effect of statins on mood and long-term reduction in total cholesterol concentration were evaluated in a randomized placebo-controlled study of n = 621 psychologically healthy adults [127]. Patients treated with simvastatin 20 mg or 40 mg for an average of 152 weeks were no more likely to have developed symptoms of MDD or anxiety or to have begun treatment with psychotropic than those who had been on placebo. In addition, intermittent use of statins was not associated with the development of MDD and BD in a retrospective cohort study of 13,626 statin users [128]. On the other hand, another study found an association between statin exposure and a lower rate of psychiatric hospitalization in BD patients [129]. Statins also have potent anti-inflammatory and anti-oxidative properties that may contribute to their effects on cardiovascular risk [12,130]. BD has been described as a multi-systemic inflammatory disease [131], and inflammation and oxidative stress are non-specific biological markers of BD [132,133]. To date, three human clinical trials have evaluated the effects of statins in BD. Since lovastatin has been shown to improve symptoms of MDD [58], it might be hypothesized that lovastatin should exacerbate mania in BD. To investigate this, lovastatin was added as an adjuvant to lithium for treating the manic phase of patients with BD in a randomized, placebo-controlled, double-blinded clinical trial [134]. Lovastatin was initiated at a dose of 10 mg/day and titrated up to 30 mg/day over one week. Clinical assessment after two and then four weeks of treatment demonstrated no significant difference in score using the Young Mania Rating Scale (YMRS) between lovastatin and placebo groups, and with no serious adverse effects. These results suggest that the anti-depressive effects of lovastatin do not exacerbate the manic phase of BD. The effects of atorvastatin on cognition and mood were also examined through a double-blind placebo-controlled RCT in 60 patients with BD and major depressive disorder (MDD) [135]. Administration of 20 mg/day atorvastatin for 12 weeks did not improve global cognition in these patients. Furthermore, the use of atorvastatin was not associated with less MDD relapse, mania relapse, mood episode relapse. However, statin use appeared to be safe in the study population. Cognitive dysfunction is another characteristic of BD, especially in elderly subjects [136,137]. There are no existing pharmacological treatments, but it is possible that statins may show some promise. Statins have been associated with reduced cognitive decline [130] and less adverse white matter changes [138], and it is well known that low circulating LDL cholesterol levels are generally protective. A cross-sectional study assessed the association between statin use and cognition in 143 older euthymic participants with BD [139]. There was little difference between statin users and non-users for cognitive outcomes in tests of language, memory, executive function, and visual-motor ability. The type and dose of statin were not elucidated from the data. However, it is possible that statin use for treating cardiovascular diseases may not have clinically important adverse associations with cognition in older BD patients, who may have pre-morbid cognitive decline. RCTs designed to determine the effects of statins on BD are limited. None of these RCTs demonstrated severe adverse effects following statin administration [127,134,135]. Long-term use of simvastatin 20 or 40 mg/day was not associated with any alterations in mood compared with placebo [127]. Additionally, four weeks of treatment with 30 mg/day lovastatin combined with lithium could not decrease mania symptoms more than lithium alone [134]. Moreover, 12 weeks of 20 mg/day atorvastatin did not result in less depressive, manic episode relapses versus placebo in BD patients [135]. A summary of these trials is given in Table 5. Therefore, while statins may not be associated with negative psychiatric events in BD, the potential for statins to play a role in improving mania or depressive episodes of BD needs further investigation.

## 8. Delirium and Statins

### 8.1. Delirium Definition

Delirium is recognized by brain dysfunction and cognitive impairment [140]. The incidence of delirium is increased after surgery and intensive-care stay [141]. The exact pathophysiological mechanism of long-term cognitive impairment in patients remains unknown, but it is believed that delirium is associated with neuroinflammation [142]. In addition, oxidative damage and neuronal apoptosis are increased in the brains of patients suffering from delirium [143]. The increase in serum inflammatory markers, such as IL-1β, and CNS inflammation is observed in delirious patients [144,145], and it has been shown that inflammatory markers are increased in the cerebrospinal fluid of patients with delirium [145]. A systemic inflammation resulting from severe sepsis may also cause patients to develop delirium [146]. No truly effective pharmacological agents are available for the prevention and treatment of delirium in hospitalized patients.

### 8.2. Evidence from Clinical Trials

A preclinical study showed that statins reduce the inflammation caused by severe sepsis, decrease the neural oxidative damage, and protect the brain from cognitive impairment [147]. Clinical trials have shown that statins protect patients undergoing cardiac and vascular surgery from delirium [148,149]. More importantly, statins reduced the risk of delirium in hospitalized ICU patients possibly through the reduction of systemic inflammation [150,151]. Statins, as anti-inflammatory agents, may protect the brain from neuroinflammation, oxidative damage, apoptosis, and ischemia. It is shown that statins decrease the expression of toll-like receptors (TLRs) involved in the trigger of inflammation. They also reduce the production of inflammatory molecules, such as TNF-α and IL-β [152]. Statins appear to have neuroprotective effects, as they preserve postoperative memory and increase the survival and function of neurons in brain injury [153]. As statins have putative anti-inflammatory activities, they may reduce the risk of delirium in critically ill patients. In a multi-center RCT, Needham et al. evaluated that the effect of 20 mg daily rosuvastatin on the incidence of delirium in patients with sepsis-associated acute respiratory distress syndrome. However, they showed that the administration of rosuvastatin for up to 28 days was not able to reduce the risk of delirium in such patients. A 12-month follow up showed no significant difference in cognitive impairment between intervention and placebo groups [146]. The same result is shown in another study conducted by Page and colleagues. In this modifying delirium using simvastatin (MoDUS) trial, they showed that 80 mg daily simvastatin did not reduce the risk of delirium and coma in critically ill patients undergoing mechanical ventilation [154]. In a retrospective single-center study, Lee et al. showed that the use of preoperative statins decreased the incidence of delirium in patients who underwent vascular surgery. However, preoperative statins did not reduce mortality or hospitalization time [155]. A summary of these trials is given in Table 6. A meta-analysis by Vallabhajosyula et al. showed that statins have no significant effects in reducing the symptoms of delirium [156]. Due to the limited number of published studies and contradictory findings, it is difficult to fully evaluate the effect of statins on preventing or treating delirium in critically ill patients.

## 9. Autism and Statins

### 9.1. Autism Definition

Autism spectrum disorder (ASD) is a developmental disorder that affects both communication and behavior, impacting everyday activities, including education and social interaction [157]. The primary treatment of autism is non-pharmacological intervention consisting of specialized education, physical therapy, occupational therapy, speech and language therapy, and behavioral therapy. Some individuals with symptoms of irritability and aggression benefit from pharmacological treatments, such as risperidone and aripiprazole. Neurobiological findings in autism reveal impaired excitatory/inhibitory balance of neurotransmission, lipid metabolism, and immune/inflammatory responses, and therefore, there is a basis for statins to be investigated as an adjuvant treatment although the possible mechanisms of action of statins in treating symptoms and behaviors commonly seen in autism is not yet elucidated. Statins inhibit the synthesis of cholesterol and downstream isoprenoids in the mevalonate pathway [126,158], and it has been shown that cholesterol synthesis is altered in autism [159]. Statins also have anti-inflammatory and immune-modulatory effects through the reduction of lipid peroxidation, production of reactive oxygen species, and NO [160], while increased oxidative damage and reactive oxygen species are detected in the brains of individuals with autism [161]. In addition, statins reduce the capability of leukocyte infiltration, interfere with antigen presentation to T cells, decrease pro-inflammatory cytokines, increase anti-inflammatory cytokines, and inhibit microglial activation [158]. There is evidence of immune/inflammatory changes in autism. Variations in genes coding for cell surface proteins result in increased production of pro-inflammatory cytokines in brain specimens and cerebrospinal fluid, decreasing the anti-inflammatory cytokines, and improved microglial and astrocytic activation [160]. Evidence of brain-specific patterns of increases in oxidative stress suggests a strong role for immune/inflammatory factors in autism [161], consisting of raised peripheral pro-inflammatory cytokines, decreased levels of peripheral anti-inflammatory cytokines, and defective systemic cell-mediated immunity [162].

### 9.2. Evidence from Clinical Trials

Few human clinical trials have, to date, evaluated the effect of statins in autism disorders. A double-blind, placebo-controlled RCT was the first report of positive effects of statins as adjunctive therapy to risperidone on irritability symptoms in 66 children with autism disorder [163]. Simvastatin was administered at 20 mg/day to children <10 years of age and 40 mg/day for those ≥10 years of age for 10 weeks. The difference in the Aberrant Behavior Checklist—Community (ABC-C) scale, including lethargy/social withdrawal, stereotypic behavior, and inappropriate speech, irritability, and hyperactivity/noncompliance subscale score changes, was measured. The only significant differences were in the reduction of the ABC-C scale irritability and hyperactivity/noncompliance subscales scores. The strong time × treatment interactions in the ABC-C irritability and hyperactivity/noncompliance subscale scores indicate that patients in the simvastatin arm experienced higher response rates. There were no significant differences between the simvastatin and placebo arms in terms of the incidence of adverse events. Another RCT evaluated statin efficacy in autism treatment of 26 children with neurofibromatosis type 1 (NF1) [164]. Participants received placebo or simvastatin in liquid form at 0.5 mg/kg/day, and if no adverse effects were reported, the simvastatin dose was increased 1 mg/kg/day to a maximum of 30 mg/day for 12 weeks. There were no reports of severe adverse events in this study. Clinical response was observed in only 25% of the statin treatment group (compared to none of the placebo group), so no significant behavioral effect of simvastatin was observed. A summary of these trials is given in Table 7. It is worth noting that in this trial, the effects of simvastatin on the upstream processes at the cell and neural system levels were studied in detail, reflecting a relationship between gene disruption and the autism-related behavioral psychopathological outcomes known in NF1. Findings at the cellular level showed a reduction of MAPK function in peripheral lymphocytes, concordant with a cellular-level statin effect on the activation of the Ras pathway. The results of this investigation support the hypothesis that there is reduced extra-cellular water-free diffusion in NF1 and also that simvastatin may reduce intra-myelin edema and improve cellular packing. Simvastatin demonstrated specific effects in key brain areas associated with the NF1 neural pathology in previous studies that are highly associated with social impairment and autism psychopathology [165]. Neither study reported adverse effects on behaviors due to the addition of statins. Further clinical trials are warranted to understand the potential benefits of statin use in autism spectrum disorders.

## 10. Potential Adverse Effects of Statins

Statins are generally well tolerated by patients with very few adverse effects [166]. The most common side effects noticed by users include myotoxicity (myalgia, myopathy, and myositis), gastrointestinal symptoms, headache, and skin reactions [167,168,169,170]. Lipids have a key role in brain function, formation of neuron cell membranes, myelin sheath, and nerve synapses. Thereby, lowering of serum lipid levels may lead to reduction in cholesterol obtained by neurons leading to alteration of behavior, including irritability, aggression, mood swings, sleep disturbance, and suicidal thoughts [125]. Some studies report cognitive dysfunction among patients who take statin therapy, yet the causality for this correlation has not been demonstrated [171,172]. Furthermore, there are some possible but not proven links between statin use and the occurrence of hemorrhagic stroke, kidney impairment, tendon rupture, and interstitial respiratory diseases [173]. There are conflicting results from different studies in the association between statin use and occurrence of MDD. Some studies found no association (increase or decrease) in mood alteration [50,127,174,175,176], while others emphasize the possible role of statin usage in increasing of MDD scores [177]. As discussed previously, there are clinical trials outcomes suggesting statins as a potential adjuvant for better management of MDD [58,59,60]. Some researchers have hypothesized that because there is an established relationship between hyperlipidemia and MDD, administration of medication with lipid-lowering properties may also reduce the risk of depression [16,64]. Interestingly, similar findings were reported for anxiety. A retrospective study found an increased rate of aggression in consumers of statins [50,176]. Furthermore, psychiatric inpatients with low serum cholesterol are at higher risk of behavioral changes [124], whereas some studies found the positive benefit from statins administered as a protective intervention in aggressive behaviors [16,101,103]. Some observational studies report a possible association between statin use and insomnia [178]. This could be problematic and negatively affect the quality of life, as the majority of statin consumers are elderly, and around 50% of older people have insomnia [179]. In a randomized placebo-controlled clinical trial of pravastatin as an adjunctive therapy in schizophrenia patients, muscle soreness was the only notable side effect in the patients receiving pravastatin in comparison to the placebo group [84]. When simvastatin was administered at 0.5 mg/kg/day in autistic children with neurofibromatosis type 1, no adverse effects were reported [164]. Similar findings were reported when simvastatin 20–40 mg/day was added to risperidone to reduce irritability and hyperactivity in children with autism. No adverse effects were reported on behaviors due to the addition of statins [163]. Simvastatin and lovastatin as add-on therapies in patients with schizophrenia were not associated with serious side effects [80,83].

## 11. Future Prospect

Much evidence suggests that inflammation is a key factor involved in the pathogenesis of many psychiatric diseases. Statins have anti-inflammatory and anti-oxidant properties and should be considered as part of a treatment package for some neuropsychiatric disorders.

One of the limitations of this narrative review is lack of quantitative analysis, which limits the strength of our conclusions. Given the small sample size and the low statistical power to detect an efficacy outcome difference, the data assessed herein should be cautiously interpreted. Accordingly, we recommend further studies in the form of phase II clinical trials to be conducted to evaluate the possibility of beneficial effects of statins in neuropsychiatric disorders. Therefore, it is recommended that future research pay more attention to determining the efficacy of statins in neuropsychological outcomes by using different instruments and especially in specific subgroups of patients identified by pre-treatment assessments of inflammatory and lipid profiles.

## 12. Conclusions

Several studies have evaluated the effect of statins in neuropsychiatric disorders. The beneficial effect of adjunctive statin therapy in improving depressive symptoms has been demonstrated in several clinical trials. Although lipophilic statins (including simvastatin) because of better brain penetrance may have greater protective effects against MDD than hydrophilic statins (including rosuvastatin and pravastatin), more studies are needed to prove this matter. The effects of statins in reducing the psychiatric symptoms of schizophrenic patients are doubtful, but it seems that the use of lipophilic statins, such as simvastatin with sufficient dose and duration, could be effective, especially in relieving negative symptoms. The significant positive effects of statins in the treatment of anxiety disorders without any serious adverse side effects were shown in numerous studies. In OCD, BD, and delirium, limitations and contradictions in the available data make it difficult to draw conclusions on any positive effect of statins, and further investigation is needed. The positive effects of simvastatin in autism disorders have been evaluated in only a small number of clinical trials, but further studies must be performed to determine their effects properly. Therefore, further extensive, well-designed prospective clinical trials are warranted to establish any therapeutic role for statins in neuropsychiatric disorders.

## Figures and Tables

**Table 1:** Summary of clinical trials using in major depressive disorders. HDR, Hamilton Depression Rating; MDD, Major depressive disorder; RCT, Randomized Clinical Trial.

Disease	Statin Type	No. of Patients	Study Design	Dosage/Duration	Main Outcomes	References
Coronary artery bypass graft	Simvastatin and atorvastatin	46	Double-blind RCT	20 mg/day for 6 weeks	Simvastatin tended to improve depressive symptoms earlier and more effectively than did atorvastatin	[60]
MDD	Atorvastatin	60	Double-blind RCT	20 mg/day for 12 weeks	Adjuvant atorvastatin favorably affect symptoms of MDD among patients with severe MDD	[58]
MDD	Lovastatin	68	Double-blind RCT	30 mg/day for 6 weeks	Treatment group was more effective than placebo group in reduction of MDD score	[57]
Moderate to severe MDD	Simvastatin	48	Double-blind RCT	20 mg/day for 6 weeks	Simvastatin showed early improvement and response rates with more reductions in HDR scores compared to the placebo group	[59]
Youth MDD	Rosuvastatin	85	Triple-blind RCT	10 mg/day for 12 weeks	Aspirin and rosuvastatin can be considered as new adjunctive treatment options for youth MDD	[61]

**Table 2:** Summary of clinical trials using in schizophrenia. BD, bipolar disorder; TC, total cholesterol; LDL, lo-density lipoprotein; TG, triglyceride; PANSS, Positive and Negative Syndrome Scale; RCT, randomized clinical trial.

Disease	Statin Type	No. of Patients	Study Design	Dosage/Duration	Main Outcomes	References
Schizophrenia	Simvastatin	66	Double-blind RCT	40 mg daily/8 weeks	Reduction of negative symptoms of schizophrenia	[17]
Schizophrenia and Schizoaffective Disorder	Rosuvastatin	100	RCT	Unknown/3 months	Reduction of TC, LDL, and TG	[79]
Schizophrenia, bipolar psychosis, organic psychosis	Statins including atorvastatin (n = 20), fluvastatin (n = 2), rosuvastatin (n = 2), or simvastatin (n = 4)	28	Retrospective, single-center study	Atorvastatin (19 ± 8.5 mg/day), fluvastatin (80 mg/day), rosuvastatin (10 mg/day), and simvastatin (10–20 mg/day)/14–54 days	Reduction of TC, LDL, and TG.	[80]
Schizophrenia	Simvastatin	216	Two-center double-blind RCT	20 mg once daily giving rise to 40 mg once daily after 4 weeks/26 weeks	Improvement in symptoms of schizophrenia, no serious side-effects	[81]
Psychologic disorders	Statins including simvastatin, atorvastatin, pravastatin, rosuvastatin, fluvastatin, or lovastatin	46,249	Retrospective, cohort study	Unknown	A similar risk of developing psychologic disorders	[82]
Schizophrenia	Lovastatin	36	Double-blind mdd	20 mg daily/8 weeks	No changes in the PANSS score	[83]
Schizophrenia	Pravastatin	60	Pilot RCT	40 mg daily/12 weeks	Reduction of cholesterol and LDL, no changes in cognition and psychiatric scores	[84]
Schizophrenia, BD, psychosis	Statins	47 out of 144 recruited patients	Cluster RCT	Unknown/12 months	No reduction in TC	[85]

**Table 3:** Summary of clinical trials using in anxiety disorders.

Disease	Statin Type	No. of Patients	Study Design	Dosage/Duration	Main Outcomes	References
Anxiety and nonadherence to statin therapy	Unknown	1924	Prospective cohort study	Not defined	Frequent occurrence of somatic symptoms of anxiety but not psychological symptoms was found to be associated with future nonadherence to statin therapy	[101]
New-onset anxiety/depression in patients with head and neck cancer	Unknown	1632	A matched longitudinal cohort study	Not defined	Statins use for head and neck cancer patients with hyperlipidemia could decrease the risk of anxiety/depression	[102]
Anxiety and depression in patients with asthma-chronic obstructive pulmonary disease overlap syndrome	Unknown	9139	Retrospective cohort study	Not defined	The incidences of anxiety and depression were relatively low among users of statins	[103]

**Table 4:** Summary of clinical trials using in OCD. Y-BOCS, Yale–Brown Obsessive-Compulsive Symptoms; OCD, Obsessive-Compulsive Disorder; RCT, randomized clinical trial.

Disease	Statin Type	No. of Patients	Study Design	Dosage/Duration	Main Outcomes	References
OCD	Atorvastatin	26	Double-blind RCT	10 mg daily/12 weeks	Reduction of Y-BOCS scores	[121]
OCD	Atorvastatin	64	Double-blind RCT	40 mg daily/1 year	Reduction of Y-BOCS scores	[122]

**Table 5:** Summary of clinical trials using in BD. BD, bipolar disorder; MDD, major depressive disorder; RCT, randomized clinical T = trial.

Disease	Statin Type	No. of Patients	Study Design	Dosage/Duration	Main Outcomes	References
BD and MDD	Atorvastatin	60	Double-blinded, placebo-controlled RCT	Atorvastatin 20 mg/day for the 12-week duration of the study.	No significantly different between atorvastatin and placebo groups in MDD relapse or mania relapse or any mood episode relapse	[135]
BD	Unknown	143	Cross-sectional	Unknown	Statin use is not independently associated with cognitive impairment in older patients with bipolar disease	[139]
BD	Lovastatin	45	Double-blinded, placebo-controlled RCT	Started with the dose of 10 mg/day, then titrated up to 30 mg/day during one week for 4 weeks	No significant difference between groups in exacerbation or decrease the symptoms of mania in patients with BD	[134]
Psychologically healthy	Simvastatin	621	RCT	20 mg or 40 mg or followed up at an average of 152 weeks	No significant differences in the use of psychotropic medication or in reports of symptoms possibly related to mood	[127]

**Table 6:** Summary of clinical trials using in delirium. RCT, randomized clinical trial.

Disease	Statin Type	No. of Patients	Study Design	Dosage/Duration	Main Outcomes	References
Delirium	Rosuvastatin	272	Multi-center RCT	40 mg loading dose with 20 mg daily/up to 28 days	No changes in delirium incidence and cognitive impairment	[146]
Delirium	Simvastatin	142	Double-blind RCT	80 mg daily/up to 28 days	No reduction in delirium incidence and coma	[154]
Delirium	Preoperative statins	1132	Retrospective, single-center study	Unknown	A decrease in delirium incidence	[155]

**Table 7:** Summary of clinical trials using in autism spectrum disorders. ABC-C, Aberrant Behavior Checklist–Community; RCT, randomized clinical trial.

Disease	Statin Type	No. of Patients	Study Design	Dosage/Duration	Main Outcomes	References
Autism	Simvastatin	66	Double-blind RCT	Children <10 years: 20 mg/day Children >10 years: 40 mg/day for 10 weeks	Significant differences in change of the ABC-C scale irritability and hyperactivity/noncompliance. No significant difference in lethargy/social withdrawal, stereotypic behavior, and inappropriate speech subscale	[163]
Neurofibromatosis Type 1-Autism	Simvastatin	26	A single-site triple-blind (clinician, family, assessor) placebo-controlled RCT	Simvastatin or placebo in liquid preparation at 0.5 mg/kg in a single daily dose, then increased to 1 mg/kg/day to a maximum of 30 mg/day for 12 weeks	No significant difference in clinical response. Multiparametric imaging suggests possible simvastatin effects in brain areas previously associated with NF1 pathophysiology and the social brain network	[164]

## Data Availability

There is no raw data associated with this review article.

## References

[1] O’Neil A., Sanna L., Redlich C., Sanderson K., Jacka F., Williams L.J., Pasco J.A., Berk M. (2012). The impact of statins on psychological wellbeing: A systematic review and meta-analysis. BMC Med..

[2] Bellosta S., Bernini F., Paoletti R., Corsini A. (2000). Non-lipid-related effects of statins. Ann. Med..

[3] Sahebkar A., Watts G.F. (2013). New therapies targeting apoB metabolism for high-risk patients with inherited dyslipidaemias: What can the clinician expect?. Cardiovasc. Drugs Ther..

[4] Sahebkar A., Watts G.F. (2013). New LDL-cholesterol lowering therapies: Pharmacology, clinical trials, and relevance to acute coronary syndromes. Clin. Ther..

[5] Bahrami A., Parsamanesh N., Atkin S.L., Banach M., Sahebkar A. (2018). Effect of statins on toll-like receptors: A new insight to pleiotropic effects. Pharmacol. Res..

[6] Ferretti G., Bacchetti T., Sahebkar A. (2015). Effect of statin therapy on paraoxonase-1 status: A systematic review and meta-analysis of 25 clinical trials. Prog. Lipid Res..

[7] Gorabi A.M., Kiaie N., Pirro M., Bianconi V., Jamialahmadi T., Sahebkar A. (2021). Effects of statins on the biological features of mesenchymal stem cells and therapeutic implications. Heart Fail. Rev..

[8] Parizadeh S.M.R., Azarpazhooh M.R., Moohebati M., Nematy M., Ghayour-Mobarhan M., Tavallaie S., Rahsepar A.A., Amini M., Sahebkar A., Mohammadi M. (2011). Simvastatin therapy reduces prooxidant-antioxidant balance: Results of a placebo-controlled cross-over trial. Lipids.

[9] Sahebkar A., Serban C., Mikhailidis D.P., Undas A., Lip G.Y.H., Muntner P., Bittner V., Ray K.K., Watts G.F., Hovingh G.K. (2015). Association between statin use and plasma d-dimer levels: A systematic review and meta-analysis of randomised controlled trials. Thromb. Haemost..

[10] Vahedian-Azimi A., Mohammadi S.M., Beni F.H., Banach M., Guest P.C., Jamialahmadi T., Sahebkar A. (2021). Improved COVID-19 ICU admission and mortality outcomes following treatment with statins: A systematic review and meta-analysis. Arch. Med. Sci..

[11] Yildirir A., Muderrisoglu H. (2004). Non-lipid effects of statins: Emerging new indications. Curr. Vasc. Pharmacol..

[12] Kim S.-W., Kang H.-J., Jhon M., Kim J.-W., Lee J.-Y., Walker A.J., Agustini B., Kim J.M., Berk M. (2019). Statins and inflammation: New therapeutic opportunities in psychiatry. Front. Psychiatry.

[13] Hammond T.R., Marsh S.E., Stevens B. (2019). Immune signaling in neurodegeneration. Immunity.

[14] Milenkovic V.M., Stanton E.H., Nothdurfter C., Rupprecht R., Wetzel C.H. (2019). The role of chemokines in the pathophysiology of major depressive disorder. Int. J. Mol. Sci..

[15] Yan Q. (2018). Neuroimmune imbalances and Yin-Yang dynamics in stress, anxiety, and depression. Psychoneuroimmunology.

[16] Young-Xu Y., Chan K.A., Liao J.K., Ravid S., Blatt C.M. (2003). Long-term statin use and psychological well-being. J. Am. Coll. Cardiol..

[17] Tajik-Esmaeeli S., Moazen-Zadeh E., Abbasi N., Shariat S.V., Rezaei F., Salehi B., Akhondzadeh S. (2017). Simvastatin adjunct therapy for negative symptoms of schizophrenia: A randomized double-blind placebo-controlled trial. Int. Clin. Psychopharmacol..

[18] Zandi P., Sparks D., Khachaturian A., Tschanz J., Norton M., Steinberg M., Welsh-Bohmer K.A., Breitner J.C., Cache County Study investigators (2005). Cache County Investigators Do statins reduce risk of incident dementia and Alzheimer’s disease. Arch. Gen. Psychiatry.

[19] Sparks D.L., Kryscio R.J., Sabbagh M.N., Connor D.J., Sparks L.M., Liebsack C. (2008). Reduced risk of incident AD with elective statin use in a clinical trial cohort. Curr. Alzheimer Res..

[20] Zissimopoulos J.M., Barthold D., Brinton R.D., Joyce G. (2017). Sex and race differences in the association between statin use and the incidence of Alzheimer disease. JAMA Neurol..

[21] Zamrini E., McGwin G., Roseman J.M. (2004). Association between statin use and Alzheimer’s disease. Neuroepidemiology.

[22] Paoletti R., Bolego C., Cignarella A. (2005). Lipid and non-lipid effects of statins. Atheroscler. Diet Drugs.

[23] Mora S., Ridker P.M. (2006). Justification for the Use of Statins in Primary Prevention: An Intervention Trial Evaluating Rosuvastatin (JUPITER)—Can C-reactive protein be used to target statin therapy in primary prevention?. Am. J. Cardiol..

[24] Jašović-Gašić M. (2015). Is treatment-resistance in psychiatric disorders a trap for polypharmacy?. Psychiatr. Danub..

[25] Stertz L., Magalhães P.V.S., Kapczinski F. (2013). Is bipolar disorder an inflammatory condition? The relevance of microglial activation. Curr. Opin. Psychiatry.

[26] Barger S.W., Basile A.S. (2001). Activation of microglia by secreted amyloid precursor protein evokes release of glutamate by cystine exchange and attenuates synaptic function. J. Neurochem..

[27] Takaki J., Fujimori K., Miura M., Suzuki T., Sekino Y., Sato K. (2012). L-glutamate released from activated microglia downregulates astrocytic L-glutamate transporter expression in neuroinflammation: The ‘collusion’hypothesis for increased extracellular L-glutamate concentration in neuroinflammation. J. Neuroinflammation.

[28] Réus G.Z., Fries G.R., Stertz L., Badawy M., Passos I., Barichello T., Kapczinski F., Quevedo J. (2015). The role of inflammation and microglial activation in the pathophysiology of psychiatric disorders. Neuroscience.

[29] Tanaka M., Tóth F., Polyák H., Szabó Á., Mándi Y., Vécsei L. (2021). Immune Influencers in Action: Metabolites and Enzymes of the Tryptophan-Kynurenine Metabolic Pathway. Biomedicines.

[30] Link A., Ayadhi T., Böhm M., Nickenig G. (2006). Rapid immunomodulation by rosuvastatin in patients with acute coronary syndrome. Eur. Heart J..

[31] Mayer C., Gruber H., Landl E., Pailer S., Scharnagl H., Truschnig-Wilders M., März W. (2007). Rosuvastatin reduces interleukin-6-induced expression of C-reactive protein in human hepatocytes in a STAT3-and C/EBP-dependent fashion. Int. J. Clin. Pharmacol. Ther..

[32] Williams L.J., Pasco J.A., Mohebbi M., Jacka F.N., Stuart A.L., Venugopal K., O’Neil A., Berk M. (2016). Statin and aspirin use and the risk of mood disorders among men. Int. J. Neuropsychopharmacol..

[33] Kohler O., Petersen L., Mors O., Gasse C. (2015). Inflammation and depression: Combined use of selective serotonin reuptake inhibitors and NSAIDs or paracetamol and psychiatric outcomes. Brain Behav..

[34] Abbasi S.-H., Hosseini F., Modabbernia A., Ashrafi M., Akhondzadeh S. (2012). Effect of celecoxib add-on treatment on symptoms and serum IL-6 concentrations in patients with major depressive disorder: Randomized double-blind placebo-controlled study. J. Affect. Disord..

[35] Muller N., Schwarz M.J., Dehning S., Douhe A., Cerovecki A., Goldstein-Miller B., Spellmann I., Hetzel G., Maino K., Kleindienst N. (2006). The cyclooxygenase-2 inhibitor celecoxib has therapeutic effects in major depression: Results of a double-blind, randomized, placebo controlled, add-on pilot study to reboxetine. Mol. Psychiatry.

[36] Raison C.L., Rutherford R.E., Woolwine B.J., Shuo C., Schettler P., Drake D.F., Haroon E., Miller A.H. (2013). A randomized controlled trial of the tumor necrosis factor antagonist infliximab for treatment-resistant depression: The role of baseline inflammatory biomarkers. JAMA Psychiatry.

[37] Dean O.M., Maes M., Ashton M., Berk L., Kanchanatawan B., Sughondhabirom A., Tangwongchai S., Ng C., Dowling N., Malhi G.S. (2014). Protocol and rationale-the efficacy of minocycline as an adjunctive treatment for major depressive disorder: A double blind, randomised, placebo controlled trial. Clin. Psychopharmacol. Neurosci..

[38] Berk M., Dean O.M., Cotton S.M., Jeavons S., Tanious M., Kohlmann K., Hewitt K., Moss K., Allwang C., Schapkaitz I. (2014). The efficacy of adjunctive N-acetylcysteine in major depressive disorder: A double-blind, randomized, placebo-controlled trial. J. Clin. Psychiatry.

[39] Kohler O., Benros M.E., Nordentoft M., Farkouh M.E., Iyengar R.L., Mors O., Krogh J. (2014). Effect of anti-inflammatory treatment on depression, depressive symptoms, and adverse effects: A systematic review and meta-analysis of randomized clinical trials. JAMA Psychiatry.

[40] Weitz-Schmidt G. (2002). Statins as anti-inflammatory agents. Trends Pharmacol. Sci..

[41] American Psychiatric Association (2009). Practice Guideline for the Treatment of Patients with Major Depressive Disorder (3rd). http://psychiatryonline.org/guidelines.aspx.

[42] Trivedi M.H., Rush A.J., Wisniewski S.R., Nierenberg A.A., Warden D., Ritz L., Norquist G., Howland R.H., Lebowitz B., McGrath P.J. (2006). Evaluation of outcomes with citalopram for depression using measurement-based care in STAR* D: Implications for clinical practice. Am. J. Psychiatry.

[43] Benros M.E., Waltoft B.L., Nordentoft M., Ostergaard S.D., Eaton W.W., Krogh J., Mortensen P.B. (2013). Autoimmune diseases and severe infections as risk factors for mood disorders: A nationwide study. JAMA Psychiatry.

[44] Muller N. (2014). Immunology of major depression. Neuroimmunomodulation.

[45] Wium-Andersen M.K., Orsted D.D., Nordestgaard B.G. (2013). Association between elevated plasma fibrinogen and psychological distress, and depression in 73,367 individuals from the general population. Mol. Psychiatry.

[46] Salagre E., Fernandes B.S., Dodd S., Brownstein D.J., Berk M. (2016). Statins for the treatment of depression: A meta-analysis of randomized, double-blind, placebo-controlled trials. J. Affect. Disord..

[47] Kohler O., Gasse C., Petersen L., Ingstrup K.G., Nierenberg A.A., Mors O., Østergaard S.D. (2016). The effect of concomitant treatment with SSRIs and statins: A population-based study. Am. J. Psychiatry.

[48] Santanello N.C., Barber B.L., Applegate W.B., Elam J., Curtis C., Hunninghake D.B., Gordon D.J. (1997). Effect of pharmacologic lipid lowering on health-related quality of life in older persons: Results from the Cholesterol Reduction in Seniors Program (CRISP) Pilot Study. J. Am. Geriatr. Soc..

[49] Hyyppa M.T., Kronholm E., Virtanen A., Leino A., Jula A. (2003). Does simvastatin affect mood and steroid hormone levels in hypercholesterolemic men? A randomized double-blind trial. Psychoneuroendocrinology.

[50] Collins R., Reith C., Emberson J., Armitage J., Baigent C., Blackwell L., Blumenthal R., Danesh J., Smith G.D., DeMets D. (2016). Interpretation of the evidence for the efficacy and safety of statin therapy. Lancet.

[51] Zhang X., Norris S.L., Gregg E.W., Cheng Y.J., Beckles G., Kahn H.S. (2005). Depressive symptoms and mortality among persons with and without diabetes. Am. J. Epidemiol..

[52] Otte C., Zhao S., Whooley M.A. (2012). Statin use and risk of depression in patients with coronary heart disease: Longitudinal data from the heart and soul study. J. Clin. Psychiatry.

[53] Parsaik A.K., Singh B., Hassan M.M., Singh K., Mascarenhas S.S., Williams M.D., Lapid M.I., Richardson J.W., West C.P., Rummans T.A. (2014). Statins use and risk of depression: A systematic review and meta-analysis. J. Affect. Disord..

[54] Köhler-Forsberg O., Gasse C., Berk M., Østergaard S.D. (2017). Do Statins Have Antidepressant Effects?. CNS Drugs.

[55] Kessing L.V., Rytgaard H.C., Gerds T.A., Berk M., Ekstrøm C.T., Andersen P.K. (2019). New drug candidates for depression—A nationwide population-based study. Acta Psychiatr. Scand..

[56] Köhler-Forsberg O., Gasse C., Petersen L., Nierenberg A.A., Mors O., Østergaard S.D. (2019). Statin treatment and the risk of depression. J. Affect Disord..

[57] Ghanizadeh A., Hedayati A. (2013). Augmentation of fluoxetine with lovastatin for treating major depressive disorder, a randomized double-blind placebo controlled-clinical trial. Depress Anxiety.

[58] Haghighi M., Khodakarami S., Jahangard L., Ahmadpanah M., Bajoghli H., Holsboer-Trachsler E., Brand S. (2014). In a randomized, double-blind clinical trial, adjuvant atorvastatin improved symptoms of depression and blood lipid values in patients suffering from severe major depressive disorder. J. Psychiatr. Res..

[59] Gougol A., Zareh-Mohammadi N., Raheb S., Farokhnia M., Salimi S., Iranpour N., Yekehtaz H., Akhondzadeh S. (2015). Simvastatin as an adjuvant therapy to fluoxetine in patients with moderate to severe major depression: A double-blind placebo-controlled trial. J. Psychopharmacol..

[60] Abbasi S.H., Mohammadinejad P., Shahmansouri N., Salehiomran A., Beglar A.A., Zeinoddini A., Forghani S., Akhondzadeh S. (2015). Simvastatin versus atorvastatin for improving mild to moderate depression in post-coronary artery bypass graft patients: A double-blind, placebo-controlled, randomized trial. J. Affect. Disord..

[61] Quinn A.L., Dean O.M., Davey C.G., Kerr M., Harrigan S.M., Cotton S.M., Chanen A.M., Dodd S., Ratheesh A., Amminger G.P. (2018). Youth Depression Alleviation-Augmentation with an anti-inflammatory agent (YoDA-A): Protocol and rationale for a placebo-controlled randomized trial of rosuvastatin and aspirin. Early Interv. Psychiatry.

[62] Redlich C., Berk M., Williams L.J., Sundquist J., Sundquist K., Li X. (2014). Statin use and risk of depression: A Swedish national cohort study. BMC Psychiatry.

[63] Chuang C.-S., Yang T.-Y., Muo C.-H., Su H.-L., Sung F.-C., Kao C.-H. (2014). Hyperlipidemia, statin use and the risk of developing depression: A nationwide retrospective cohort study. Gen. Hosp. Psychiatry.

[64] Kim J.-M., Stewart R., Kang H.-J., Bae K.-Y., Kim S.-W., Shin I.-S., Kim J.T., Park M.S., Cho K.H., Yoon J.S. (2014). A prospective study of statin use and poststroke depression. J. Clin. Psychopharmacol..

[65] Al Badarin F.J., Spertus J.A., Gosch K.L., Buchanan D.M., Chan P.S. (2013). Initiation of statin therapy after acute myocardial infarction is not associated with worsening depressive symptoms: Insights from the Prospective Registry Evaluating Outcomes After Myocardial Infarctions: Events and Recovery (PREMIER) and Translational Research Investigating Underlying Disparities in Acute Myocardial Infarction Patients’ Health Status (TRIUMPH) registries. Am. Heart J..

[66] Glaus J., Vandeleur C.L., Lasserre A.l.M., Strippoli M.-P.F., Castelao E., Gholam-Rezaee M., Waeber G., Aubry J.M., Vollenweider P., Preisig M. (2015). Aspirin and statin use and the subsequent development of depression in men and women: Results from a longitudinal population-based study. J. Affect. Disord..

[67] Kim S.W., Bae K.Y., Kim J.M., Shin I.S., Hong Y.J., Ahn Y., Jeong M.H., Berk M., Yoon J.S. (2015). The use of statins for the treatment of depression in patients with acute coronary syndrome. Transl. Psychiatry.

[68] Dave C.V., Winterstein A.G., Park H., Cook R.L., Hartzema A.G. (2018). Comparative risk of lipophilic and hydrophilic statins on incident depression: A retrospective cohort study. J. Affect. Disord..

[69] National Institute for Health and Clinical Excellence (Great Britain) Psychosis and Schizophrenia in Adults: Treatment and Management.

[70] Miller B.J., Culpepper N., Rapaport M.H. (2014). C-reactive protein levels in schizophrenia: A review and meta-analysis. Clin. Schizophr. Relat. Psychoses.

[71] Najjar S., Pearlman D.M. (2015). Neuroinflammation and white matter pathology in schizophrenia: Systematic review. Schizophr. Res..

[72] Miller B.J., Buckley P., Seabolt W., Mellor A., Kirkpatrick B. (2011). Meta-analysis of cytokine alterations in schizophrenia: Clinical status and antipsychotic effects. Biol. Psychiatry.

[73] Keller W.R., Kum L.M., Wehring H.J., Koola M.M., Buchanan R.W., Kelly D.L. (2013). A review of anti-inflammatory agents for symptoms of schizophrenia. J. Psychopharmacol..

[74] Van Berckel B.N., Bossong M.G., Boellaard R., Kloet R., Schuitemaker A., Caspers E., Luurtsema G., Windhorst A.D., Cahn W., Lammertsma A.A. (2008). Microglia activation in recent-onset schizophrenia: A quantitative (R)-[11C] PK11195 positron emission tomography study. Biol. Psychiatry.

[75] Osborn D.P., Nazareth I., King M.B. (2007). Physical activity, dietary habits and Coronary Heart Disease risk factor knowledge amongst people with severe mental illness. Soc. Psychiatry Psychiatr. Epidemiol..

[76] Laursen T.M., Munk-Olsen T., Gasse C. (2011). Chronic somatic comorbidity and excess mortality due to natural causes in persons with schizophrenia or bipolar affective disorder. PLoS ONE.

[77] Olfson M., Marcus S.C., Corey-Lisle P., Tuomari A., Hines P., L’Italien G.J. (2006). Hyperlipidemia following treatment with antipsychotic medications. Am. J. Psychiatry.

[78] Hsu J.-H., Chien I.-C., Lin C.-H., Chou Y.-J., Chou P. (2012). Hyperlipidemia in patients with schizophrenia: A national population-based study. Gen. Hosp. Psychiatry.

[79] De M.H., Kalnicka D., Wampers M., Hanssens L., Van D.E., Scheen A., Peuskens J. (2006). treatment with rosuvastatin for severe dyslipidemia in patients with schizophrenia and schizoaffective disorder. J. Clin. Psychiatry.

[80] Ojala K., Repo-Tiihonen E., Tiihonen J., Niskanen L. (2008). Statins are effective in treating dyslipidemia among psychiatric patients using second-generation antipsychotic agents. J. Psychopharmacol..

[81] Chaudhry I.B., Husain N., Husain M.O., Hallak J., Drake R., Kazmi A., ur Rahman R., Hamirani M.M., Kiran T., Mehmood N. (2013). Ondansetron and simvastatin added to treatment as usual in patients with schizophrenia: Study protocol for a randomized controlled trial. Trials.

[82] Mansi I., Frei C.R., Pugh M.J., Mortensen E.M. (2013). Psychologic disorders and statin use: A propensity score-matched analysis. Pharmacother. J. Hum. Pharmacol. Drug Ther..

[83] Ghanizadeh A., Rezaee Z., Dehbozorgi S., Berk M., Akhondzadeh S. (2014). Lovastatin for the adjunctive treatment of schizophrenia: A preliminary randomized double-blind placebo-controlled trial. Psychiatry Res..

[84] Vincenzi B., Stock S., Borba C.P., Cleary S.M., Oppenheim C.E., Petruzzi L.J., Fan X., Copeland P.M., Freudenreich O., Cather C. (2014). A randomized placebo-controlled pilot study of pravastatin as an adjunctive therapy in schizophrenia patients: Effect on inflammation, psychopathology, cognition and lipid metabolism. Schizophr. Res..

[85] Osborn D., Burton A., Hunter R., Marston L., Atkins L., Barnes T., Blackburn R., Craig T., Gilbert H., Heinkel S. (2018). Clinical and cost-effectiveness of an intervention for reducing cholesterol and cardiovascular risk for people with severe mental illness in English primary care: A cluster randomised controlled trial. Lancet Psychiatry.

[86] Nomura I., Kishi T., Ikuta T., Iwata N. (2018). Statin add-on therapy in the antipsychotic treatment of schizophrenia: A meta-analysis. Psychiatry Res..

[87] Çakici N., Van Beveren N., Judge-Hundal G., Koola M., Sommer I. (2019). An update on the efficacy of anti-inflammatory agents for patients with schizophrenia: A meta-analysis. Psychol. Med..

[88] Kato T., Monji A., Mizoguchi Y., Hashioka S., Horikawa H., Seki Y., Kasai M., Utsumi H., Kanba S. (2011). Anti-Inflammatory properties of antipsychotics via microglia modulations: Are antipsychotics a ‘fire extinguisher’in the brain of schizophrenia?. Mini Rev. Med. Chem..

[89] Wang Q., Zengin A., Deng C., Li Y., Newell K.A., Yang G.-Y., Lu Y., Wilder-Smith E.P., Zhao H., Huang X.F. (2009). High dose of simvastatin induces hyperlocomotive and anxiolytic-like activities: The association with the up-regulation of NMDA receptor binding in the rat brain. Exp. Neurol..

[90] Yan J., Xu Y., Zhu C., Zhang L., Wu A., Yang Y., Xiong Z., Deng C., Huang X.F., Yenari M.A. (2011). Simvastatin prevents dopaminergic neurodegeneration in experimental parkinsonian models: The association with anti-inflammatory responses. PLoS ONE.

[91] Blake G.J., Ridker P.M. (2000). Are statins anti-inflammatory?. Trials.

[92] McFarland A., Davey A., Anoopkumar-Dukie S. (2017). Statins reduce lipopolysaccharide-induced cytokine and inflammatory mediator release in an in vitro model of microglial-like cells. Mediat. Inflamm..

[93] Karmaus P.W., Shi M., Perl S., Biancotto A., Candia J., Cheung F., Kotliarov Y., Young N., Fessler M.B., CHI, Consortium (2019). Effects of rosuvastatin on the immune system in healthy volunteers with normal serum cholesterol. JCI Insight.

[94] Diamantis E., Kyriakos G., Victoria Quiles-Sanchez L., Farmaki P., Troupis T. (2017). The anti-inflammatory effects of statins on coronary artery disease: An updated review of the literature. Curr. Cardiol. Rev..

[95] McFarland A.J., Anoopkumar-Dukie S., Arora D.S., Grant G.D., McDermott C.M., Perkins A.V., Davey A.K. (2014). Molecular mechanisms underlying the effects of statins in the central nervous system. Int. J. Mol. Sci..

[96] Felger J.C. (2018). Imaging the role of inflammation in mood and anxiety-related disorders. Curr. Neuropharmacol..

[97] Vogelzangs N., Beekman A., De Jonge P., Penninx B. (2013). Anxiety disorders and inflammation in a large adult cohort. Transl. Psychiatry.

[98] Quagliato L.A., Nardi A.E. (2018). Cytokine alterations in panic disorder: A systematic review. J. Affect. Disord..

[99] Hou R., Garner M., Holmes C., Osmond C., Teeling J., Lau L., Baldwin D.S. (2017). Peripheral inflammatory cytokines and immune balance in Generalised Anxiety Disorder: Case-controlled study. Brain Behav. Immun..

[100] Shrivastava S., Pucadyil T.J., Paila Y.D., Ganguly S., Chattopadhyay A. (2010). Chronic cholesterol depletion using statin impairs the function and dynamics of human serotonin1A receptors. Biochemistry.

[101] Korhonen M.J., Pentti J., Hartikainen J., Kivimäki M., Vahtera J. (2016). Somatic symptoms of anxiety and nonadherence to statin therapy. Int. J. Cardiol..

[102] Huang C.-I., Lin L.-C., Tien H.-C., Que J., Ting W.C., Chen P.-C., Wu H.M., Ho C.H., Wang J.J., Wang R.H. (2017). Hyperlipidemia and statins use for the risk of new-onset anxiety/depression in patients with head and neck cancer: A population-based study. PLoS ONE.

[103] Yeh J.-J., Syue S.-H., Lin C.-L., Hsu C.Y., Shae Z., Kao C.-H. (2019). Effects of statins on anxiety and depression in patients with asthma-chronic obstructive pulmonary disease overlap syndrome. J. Affect. Disord..

[104] Pemminati S., Colaco M.N., Patchava D., Shivaprakash G., Ullal D.S., Gopalakrishna H., Rathnakar U.P., Shenoy A.K. (2012). Role of statins in animal models of anxiety in Normo-cholesterolemic rats. J. Pharm. Res..

[105] Thomas J.M., Varkey J., Augustine B.B. (2014). Association between serum cholesterol, brain serotonin, and anxiety: A study in simvastatin administered experimental animals. Int. J. Nutr. Pharmacol. Neurol. Dis..

[106] Stein D.J. (2002). Obsessive-compulsive disorder. Lancet.

[107] Ravizza L., Barzega G., Bellino S., Bogetto F., Maina G. (1996). Drug treatment of obsessive-compulsive disorder (OCD): Long-term trial with clomipramine and selective serotonin reuptake inhibitors (SSRIs). Psychopharmacol. Bull..

[108] Decloedt E.H., Stein D.J. (2010). Current trends in drug treatment of obsessive–compulsive disorder. Neuropsychiatr. Dis. Treat..

[109] Koo M.-S., Kim E.-J., Roh D., Kim C.-H. (2010). Role of dopamine in the pathophysiology and treatment of obsessive–compulsive disorder. Expert Rev. Neurother..

[110] Ting J.T., Feng G. (2008). Glutamatergic synaptic dysfunction and obsessive-compulsive disorder. Curr. Chem. Genom..

[111] Pallanti S., Quercioli L. (2006). Treatment-refractory obsessive-compulsive disorder: Methodological issues, operational definitions and therapeutic lines. Prog. Neuro-Psychopharmacol. Biol. Psychiatry.

[112] Yang J.-w., Hu Z.-p. (2015). Neuroprotective effects of atorvastatin against cerebral ischemia/reperfusion injury through the inhibition of endoplasmic reticulum stress. Neural Regen. Res..

[113] Pathak N.N., Balaganur V., Lingaraju M.C., More A.S., Kant V., Kumar D., Kumar D., Tandan S.K. (2013). Antihyperalgesic and anti-inflammatory effects of atorvastatin in chronic constriction injury-induced neuropathic pain in rats. Inflammation.

[114] Wang Q., Ting W.L., Yang H., Wong P.T. (2005). High doses of simvastatin upregulate dopamine D1 and D2 receptor expression in the rat prefrontal cortex: Possible involvement of endothelial nitric oxide synthase. Br. J. Pharmacol..

[115] Wang Q., Tang X.N., Wang L., Yenari M.A., Ying W., Goh B.-C., Lee H.S., Wilder-Smith E.P., Wong P.T. (2006). Effects of high dose of simvastatin on levels of dopamine and its reuptake in prefrontal cortex and striatum among SD rats. Neurosci. Lett..

[116] Guimaraes F., Beijamini V., Moreira F., Aguiar D., De Lucca A. (2005). Role of nitric oxide in brain regions related to defensive reactions. Neurosci. Biobehav. Rev..

[117] Umathe S., Bhutada P., Jain N., Mundhada Y., Borkar S., Dhumal B. (2009). Role of nitric oxide in obsessive–compulsive behavior and its involvement in the anti-compulsive effect of paroxetine in mice. Nitric Oxide.

[118] Seker F., Kilic U., Caglayan B., Ethemoglu M., Caglayan A., Ekimci N., Demirci S., Dogan A., Oztezcan S., Sahin F. (2015). HMG-CoA reductase inhibitor rosuvastatin improves abnormal brain electrical activity via mechanisms involving eNOS. Neuroscience.

[119] Lin H.-C., Kang B.-H., Wan F.-J., Huang S.-T., Tseng C.-J. (2000). Reciprocal regulation of nitric oxide and glutamate in the nucleus tractus solitarii of rats. Eur. J. Pharmacol..

[120] Serra P.A., Rocchitta G., Esposito G., Delogu M.R., Migheli R., Miele E., Desole M.S., Miele M. (2001). A study on the role of nitric oxide and iron in 3-morpholino-sydnonimine-induced increases in dopamine release in the striatum of freely moving rats. Br. J. Pharmacol..

[121] Rahim F., Sayyah M. (2018). Effects of atorvastatin on treatment-resistant obsessive-compulsive disorder: A double-blind randomized trial. Psychiatr. Pol..

[122] Akouchekian S., Omranifard V., Moshfegh P., Maracy M.R., Almasi A. (2018). The Effect of Atorvastatin on Obsessive-compulsive Symptoms of Refractory Obsessive-compulsive Disorder (Add-on Therapy). Adv. Biomed. Res..

[123] While A., Keen L. (2012). The effects of statins on mood: A review of the literature. Eur. J. Cardiovasc. Nurs..

[124] Leppien E., Mulcahy K., Demler T.L., Trigoboff E., Opler L. (2018). Effects of statins and cholesterol on patient aggression: Is there a connection?. Innov. Clin. Neurosci..

[125] Tatley M., Savage R. (2007). Psychiatric adverse reactions with statins, fibrates and ezetimibe. Drug Saf..

[126] Ling Q., Tejada-Simon M.V. (2016). Statins and the brain: New perspective for old drugs. Prog. Neuro-Psychopharmacol. Biol. Psychiatry.

[127] Wardle J., Armitage J., Collins R., Wallendszus K., Keech A., Lawson A. (1996). Randomised placebo controlled trial of effect on mood of lowering cholesterol concentration. BMJ.

[128] Lilly S.M., Mortensen E.M., Frei C.R., Pugh M.J., Mansi I.A. (2014). Comparison of the risk of psychological and cognitive disorders between persistent and nonpersistent statin users. Am. J. Cardiol..

[129] Hayes J.F., Lundin A., Wicks S., Lewis G., Wong I.C., Osborn D.P., Dalman C. (2019). Association of hydroxylmethyl glutaryl coenzyme A reductase inhibitors, L-Type calcium channel antagonists, and biguanides with rates of psychiatric hospitalization and self-harm in individuals with serious mental illness. JAMA Psychiatry.

[130] Berk M., Kapczinski F., Andreazza A.C., Dean O., Giorlando F., Maes M., Yücel M., Gama C.S., Dodd S., Dean B. (2011). Pathways underlying neuroprogression in bipolar disorder: Focus on inflammation, oxidative stress and neurotrophic factors. Neurosci. Biobehav. Rev..

[131] Leboyer M., Soreca I., Scott J., Frye M., Henry C., Tamouza R., Kupfer D.J. (2012). Can bipolar disorder be viewed as a multi-system inflammatory disease?. J. Affect. Disord..

[132] Pfaffenseller B., Fries G.R., Wollenhaupt-Aguiar B., Colpo G.D., Stertz L., Panizzutti B., Magalhaes P.V., Kapczinski F. (2013). Neurotrophins, inflammation and oxidative stress as illness activity biomarkers in bipolar disorder. Expert Rev. Neurother..

[133] Ghanizadeh A., Berk M. (2013). Molecular hydrogen: An overview of its neurobiological effects and therapeutic potential for bipolar disorder and schizophrenia. Med Gas Res..

[134] Ghanizadeh A., OmraniSigaroodi M., Javadpour A., Dabbaghmanesh M.H., Shafiee S. (2014). Lovastatin as an adjuvant to lithium for treating manic phase of bipolar disorder: A 4-week, randomized, double-blind, placebo-controlled clinical trial. Depress. Res. Treat..

[135] Soh J.F., Almadani A., Beaulieu S., Rajji T., Mulsant B.H., Su C.-L., Renaud S., Mucsi I., Torres-Platas S.G., Levinson A. (2020). The effect of atorvastatin on cognition and mood in bipolar disorder and unipolar depression patients: A secondary analysis of a randomized controlled trial. J. Affect. Disord..

[136] Gildengers A., Tatsuoka C., Bialko C., Cassidy K.A., Dines P., Emanuel J., Al Jurdi R.K., Gyulai L., Mulsant B.H., Young R.C. (2013). Correlates of disability in depressed older adults with bipolar disorder. Cut. Edge Psychiatry Pract..

[137] Sajatovic M., Strejilevich S.A., Gildengers A.G., Dols A., Al Jurdi R.K., Forester B.P., Kessing L.V., Beyer J., Manes F., Rej S. (2015). A report on older-age bipolar disorder from the International Society for Bipolar Disorders Task Force. Bipolar Disord..

[138] Nadkarni N.K., Perera S., Hanlon J.T., Lopez O., Newman A.B., Aizenstein H., Elam M., Harris T.B., Kritchevsky S., Yaffe K. (2015). Statins and brain integrity in older adults: Secondary analysis of the Health ABC study. Alzheimer’s Dement..

[139] Rej S., Schulte S.W., Rajji T.K., Gildengers A.G., Miranda D., Menon M., Butters M.A., Mulsant B.H. (2018). Statins and cognition in late-life bipolar disorder. Int. J. Geriatr. Psychiatry.

[140] Casarin A., McAuley D.F., Alce T.M., Zhao X., Ely E.W., Jackson J.C., McDowell C., Agus A., Murphy L., Page V.J. (2015). Evaluating early administration of the hydroxymethylglutaryl-CoA reductase inhibitor simvastatin in the prevention and treatment of delirium in critically ill ventilated patients (MoDUS trial): Study protocol for a randomized controlled trial. Trials.

[141] Ely E., Gautam S., Margolin R., Francis J., May L., Speroff T., Truman B., Dittus R., Bernard G., Inouye S. (2001). The impact of delirium in the intensive care unit on hospital length of stay. Intensive Care Med..

[142] Cerejeira J., Firmino H., Vaz-Serra A., Mukaetova-Ladinska E.B. (2010). The neuroinflammatory hypothesis of delirium. Acta Neuropathol..

[143] Cunningham C. (2011). Systemic Inflammation and Delirium: Important Co-Factors in the Progression of Dementia. Biochem. Soc. Trans..

[144] Ritter C., Tomasi C.D., Dal-Pizzol F., Pinto B.B., Dyson A., de Miranda A.S., Comim C.M., Soares M., Teixeira A.L., Quevedo J. (2014). Inflammation biomarkers and delirium in critically ill patients. Crit. Care.

[145] Cape E., Hall R.J., van Munster B.C., de Vries A., Howie S.E., Pearson A., Middleton S.D., Gillies F., Armstrong I.R., White T.O. (2014). Cerebrospinal fluid markers of neuroinflammation in delirium: A role for interleukin-1β in delirium after hip fracture. J. Psychosom. Res..

[146] Needham D.M., Colantuoni E., Dinglas V.D., Hough C.L., Wozniak A.W., Jackson J.C., Morris P.E., Mendez-Tellez P.A., Ely E.W., Hopkins R.O. (2016). Rosuvastatin versus placebo for delirium in intensive care and subsequent cognitive impairment in patients with sepsis-associated acute respiratory distress syndrome: An ancillary study to a randomised controlled trial. Lancet Respir. Med..

[147] Alexandre P.C., Reis P.A., Joana D., de JOliveira F.M., Pamplona F.A., Siqueira L.D., Neto H.C., Bozza F.A. (2013). Atorvastatin and simvastatin protects cognitive impairment in an animal model of sepsis. Crit. Care.

[148] Katznelson R., Djaiani G.N., Borger M.A., Friedman Z., Abbey S.E., Fedorko L., Karski J., Mitsakakis N., Carroll J., Beattie W.S. (2009). Preoperative use of statins is associated with reduced early delirium rates after cardiac surgery. Anesthesiol. J. Am. Soc. Anesthesiol..

[149] Katznelson R., Djaiani G., Mitsakakis N., Lindsay T.F., Tait G., Friedman Z., Wasowicz M., Beattie W.S. (2009). Delirium following vascular surgery: Increased incidence with preoperative β-blocker administration. Can. J. Anesth./J. Can. D’anesthésie.

[150] Morandi A., Hughes C.G., Thompson J.L., Pandharipande P.P., Shintani A.K., Vasilevskis E.E., Han J.H., Jackson J.C., Laskowitz D.T., Bernard G.R. (2014). Statins and delirium during critical illness: A multicenter, prospective cohort study. Crit. Care Med..

[151] Page V.J., Davis D., Zhao X.B., Norton S., Casarin A., Brown T., Ely E.W., McAuley D.F. (2014). Statin use and risk of delirium in the critically ill. Am. J. Respir. Crit. Care Med..

[152] Niessner A., Steiner S., Speidl W.S., Pleiner J., Seidinger D., Maurer G., Goronzy J.J., Weyand C.M., Kopp C.W., Huber K. (2006). Simvastatin suppresses endotoxin-induced upregulation of toll-like receptors 4 and 2 in vivo. Atherosclerosis.

[153] Wang H., Lynch J.R., Song P., Yang H.-J., Yates R.B., Mace B., Warner D.S., Guyton J.R., Laskowitz D.T. (2007). Simvastatin and atorvastatin improve behavioral outcome, reduce hippocampal degeneration, and improve cerebral blood flow after experimental traumatic brain injury. Exp. Neurol..

[154] Page V.J., Casarin A., Ely E.W., Zhao X.B., McDowell C., Murphy L., McAuley D.F. (2017). Evaluation of early administration of simvastatin in the prevention and treatment of delirium in critically ill patients undergoing mechanical ventilation (MoDUS): A randomised, double-blind, placebo-controlled trial. Lancet Respir. Med..

[155] Lee D.-S., Lee M.Y., Park C.-M., Kim D.-I., Kim Y.-W., Park Y.-J. (2018). Preoperative statins are associated with a reduced risk of postoperative delirium following vascular surgery. PLoS ONE.

[156] Vallabhajosyula S., Kanmanthareddy A., Erwin P.J., Esterbrooks D.J., Morrow L.E. (2017). Role of statins in delirium prevention in critical ill and cardiac surgery patients: A systematic review and meta-analysis. J. Crit. Care.

[157] American Psychiatric Association (2013). Diagnostic and Statistical Manual of Mental Disorders (DSM-5^®^).

[158] van der Most P.J., Dolga A.M., Nijholt I.M., Luiten P.G., Eisel U.L. (2009). Statins: Mechanisms of neuroprotection. Prog. Neurobiol..

[159] Wang H. (2014). Lipid rafts: A signaling platform linking cholesterol metabolism to synaptic deficits in autism spectrum disorders. Front. Behav. Neurosci..

[160] Young A.M., Chakrabarti B., Roberts D., Lai M.-C., Suckling J., Baron-Cohen S. (2016). From molecules to neural morphology: Understanding neuroinflammation in autism spectrum condition. Mol. Autism.

[161] Rossignol D.A., Frye R.E. (2014). Evidence linking oxidative stress, mitochondrial dysfunction, and inflammation in the brain of individuals with autism. Front. Physiol..

[162] Estes M.L., McAllister A.K. (2015). Immune mediators in the brain and peripheral tissues in autism spectrum disorder. Nat. Rev. Neurosci..

[163] Moazen-Zadeh E., Shirzad F., Karkhaneh-Yousefi M.-A., Khezri R., Mohammadi M.-R., Akhondzadeh S. (2018). Simvastatin as an adjunctive therapy to risperidone in treatment of autism: A randomized, double-blind, placebo-controlled clinical trial. J. Child Adolesc. Psychopharmacol..

[164] Stivaros S., Garg S., Tziraki M., Cai Y., Thomas O., Mellor J., Morris A.A., Jim C., Szumanska-Ryt K., Parkes L.M. (2018). Randomised controlled trial of simvastatin treatment for autism in young children with neurofibromatosis type 1 (SANTA). Mol. Autism.

[165] Johnson M.H., Griffin R., Gergely Csibra H.H., Farroni T., de Haan M., Tucker L.A., BARON–COHEN S.I., Richards J. (2005). The emergence of the social brain network: Evidence from typical and atypical development. Dev. Psychopathol..

[166] Ramkumar S., Raghunath A., Raghunath S. (2016). Statin therapy: Review of safety and potential side effects. Acta Cardiol. Sin..

[167] Pedro-Botet J., Núñez-Cortés J.M., Flores J., Rius J. (2015). Muscle symptoms related with statin therapy in general practice. Atherosclerosis.

[168] Buettner C., Burstein R. (2015). Association of statin use and risk for severe headache or migraine by serum vitamin D status: A cross-sectional population-based study. Cephalalgia.

[169] Vesza Z., Pires C., da Silva P.M. (2018). Statin-related lichenoid dermatosis: An uncommon adverse reaction to a common treatment. Eur. J. Case Rep. Intern. Med..

[170] Smith I.B., Schmidt R., Mansi I. (2017). Are Statins Associated with Upper Gastrointestinal Symptoms?. Gastroenterology.

[171] Schultz B.G., Patten D.K., Berlau D.J. (2018). The role of statins in both cognitive impairment and protection against dementia: A tale of two mechanisms. Transl. Neurodegener..

[172] Ott B.R., Daiello L.A., Dahabreh I.J., Springate B.A., Bixby K., Murali M., Trikalinos T.A. (2015). Do statins impair cognition? A systematic review and meta-analysis of randomized controlled trials. J. Gen. Intern. Med..

[173] Thompson P.D., Panza G., Zaleski A., Taylor B. (2016). Statin-associated side effects. J. Am. Coll. Cardiol..

[174] Muldoon M.F., Barger S.D., Ryan C.M., Flory J.D., Lehoczky J.P., Matthews K.A., Manuck S.B. (2000). Effects of lovastatin on cognitive function and psychological well-being. Am. J. Med..

[175] Stewart R.A., Sharples K.J., North F.M., Menkes D.B., Baker J., Simes J. (2000). Long-term assessment of psychological well-being in a randomized placebo-controlled trial of cholesterol reduction with pravastatin. Arch. Intern. Med..

[176] Olson M.B., Kelsey S.F., Matthews K.A., Bairey Merz C.N., Eteiba W., McGorray S.P., Cornell C.E., Vido D.A., Muldoon M.F. (2008). Lipid-lowering medication use and aggression scores in women: A report from the NHLBI-sponsored WISE study. J. Women’s Health.

[177] Agostini J.V., Tinetti M.E., Han L., McAvay G., Foody J.M., Concato J. (2007). Effects of statin use on muscle strength, cognition, and depressive symptoms in older adults. J. Am. Geriatr. Soc..

[178] Tuccori M., Lapi F., Testi A., Coli D., Moretti U., Vannacci A., Motola D., Salvo F., Rivolta A.L., Blandizzi C. (2008). Statin-associated psychiatric adverse events. Drug Saf..

[179] Kamel N.S., Gammack J.K. (2006). Insomnia in the elderly: Cause, approach, and treatment. Am. J. Med..

